# Qualitative and quantitative prey requirements of two aphidophagous coccinellids, *Adalia tetraspilota* and *Hippodamia variegata*

**DOI:** 10.1093/jis/14.1.72

**Published:** 2014-01-01

**Authors:** Mohd Abas Shah, Akhtar Ali Khan

**Affiliations:** 1 Division of Entomology, Sher-e-Kashmir University of Agricultural Sciences and Technology of Kashmir, Shalimar, Srinagar-190 025, India

**Keywords:** biology, essential prey, immature survival, prey density

## Abstract

The suitability of two prey species, *Aphis pomi* De Geer (Hemiptera: Aphididae) and *Brevicoryne brassicae* (L.), for two generalist aphidophagous coccinellids, *Adalia tetraspilota* (Hope) (Cole- optera: Coccinellidae) and *Hippodamia variegata* (Goeze), at various abundance levels was investigated under laboratory conditions. While both *A. pomi* and *B. brassicae* were found to be suitable, the predators performed better when feeding upon *B. brassicae* . The prey densities affected the developmental parameters of the two predators appreciably. Optimal growth and development was noted in the prey density range of 40–80 aphids per day per predator. Both species and abundance levels of prey significantly affected the larval period of the two predators. Appreciable variation in survivorship of larvae, prepupal and pupal period, and adult weight was noted by varying the prey species and prey abundance. Longer reproductive period (oviposition period) and shorter non-reproductive periods (preoviposition and postoviposition periods) were noted for females that fed on *B. brassicae* as compared to those that fed on *A. pomi* . Reproductive output was appreciably higher for females that fed on *B. brassicae* , and the fecundity decreased drastically under food shortage.

## Introduction

Ladybird beetles (Coccinellidae: Coleoptera) are important predators in natural and agricultural habitats and prey upon many economically important pests, including aphids, mealy bugs, scale insects, thrips, leaf hoppers, mites, and other soft bodied insects ( Dixon 2000 ; Khan et al. 2009 ). The predator species feed either on aphids or coccids, with a few feeding on both types of prey ( Weber and Lundgren 2009 ). Owing to their seasonal synchrony with specific preys, high foraging performance, and high reproductive efficiency, the coccinellids have the potential to be effectively employed in integrated pest management programmes ( Lanzoni et al. 2004 ). In addition to many attempts to use them in classical biological control, some being highly successful, ladybeetles are also being translo- cated or mass produced and released for the control of various pests ( Pu 1976 ; Singh 2004 ). Lately, the role of generalist predators such as ladybeetles is being recognized in conservation biological control through conservation and enhancement techniques ( Symondson et al. 2002 ; Obrycki et al. 2009 ).

Life parameters, such as rate of development, reproductive output, etc., are dependent on various biotic and abiotic environmental factors and their interaction with genetic factors. Among the biotic factors, food (quality and abundance) is perhaps the most influential factor ( Jervis et al. 2005 ). As such, preys of ladybeetles are categorized into essential, alternative, and rejected prey, on the basis of quantitative data on developmental parameters, viz. rate of development, survival, and reproductive capacity. The studies on effects of prey species on various life parameters of coccinellids have revealed many important aspects of their nutritional ecology ( Hodek and Honek 1996 ).

*Adalia tetraspilota* (Hope) and *Hippodamia (Adonia) variegata* (Goeze) are the predominant species of coccinellids in agroecosystems of Kashmir Valley, India ( Khan et al. 2007a ). *H. variegata* originated in the Palearctic region ( Gordon 1987 ) and is a widespread predator of aphids in many parts of the world ( Franzmann 2002 ). This species is considered the most important natural enemy of aphids in many countries, including Bulgaria, Ukraine, Italy, India, and Turkmenistan ( Kontodimas and Stathas 2005 ). In China, it is one of the most common species in wheat, tobacco, cotton, vegetable, and orchard ecosystems ( Yang et al. 1997 ). *A. tetraspilota* is the most abundant predatory coccinellid in Kashmir and has been observed feeding on *Aphis pomi* De Geer *, Myzus persicae* Harris *, Lipaphis erysimi* (Kaltenbach) *, Brevicoryne brassicae* (L.), *Aphis fabae* Scopoli, and *Aphis craccivora* Koch ( Khan et al. 2009 ). In Pakistan, it has been reported in Chitral Town and Drasan ( Khan et al. 2007b ). This species has also been reported from Murree (Pakistan) feeding on *Adelges* spp. and *Quadraspidiotus perni- ciosus* (Comstock) ( Irshad 2001 ) and from Nepal ( Canepari 1997 ). A lot of research attention has been directed towards the study of the biology, functional response, life table parameters, and influence of host plants on *H. variegata* ( Wu et al. 2010 ); however, no reports exist regarding the quantitative prey requirements of the predator. Similarly, no reports regarding any biological or ecological aspect of *A. tetraspilota* were found. Hence the suitability of two common aphid species, *Aphis pomi* De Geer (Hemiptera: Aphididae), infesting apple and related pome fruits, and *Brevicoryne brassicae* (L.), infesting various cruciferous vegetables in Kashmir Valley, for the two coccinellid predators in question was investigated. The two preys were supplied at various densities to elucidate the quantitative requirements for optimal growth and development of the ladybeetles.

## Materials and Methods

### Insect rearing

To rear the predator coccinellids, aphid colonies were maintained in cages (18 x 18 x 18 cm) in the laboratory on fresh twigs of apple ( *A. pomi* ) and potted seedlings of kale ( *B. brassicae* ). The colonies were collected from pesticide-free apple orchards and vegetable fields on the Sher-e-Kashmir University campus. Ladybeetle cultures were initiated by collecting newly emerged overwintering adults of the respective coccinellid species. The adults of the two coccinellid species were maintained in plastic jars (height 20 cm, diameter 15 cm) with an abundant supply of prey from the aphid colonies until oviposition. The rearing jars were provided with crumpled paper to act as oviposition sites. The eggs laid were collected every 24 hours, transferred to Petri dishes, and allowed to hatch. All the cultures were maintained at a temperature of 25 ± 2°C and 65 ± 5% relative humidity with a photoperiod of 14:10 L:D in controlled environment rooms. Newly emerged larvae of both the predators were taken from the stock cultures and fed an *ad libitum* supply of aphids ( *A. pomi* and *B. brassicae* ). The mixed diet was provided to avoid food adaptation ( Rana et al. 2002 ). Adults from this stock were sexed, and the eggs thereof were incubated. The 1st instar larvae were used for the proposed studies. Newly emerged larvae were fed separately with 3rd or 4th instar nymphs of the two aphid species, each at five abundance levels, i.e., 10, 20, 40, 80, and 160 aphids per predator per day. The aphids fed on leaves of the respective host plants in Petri dishes. Each treatment was replicated 10 times for all parameters with at least five individuals in each replication

### Development and survival

A cohort of 50 newly emerged larvae was studied, and the durations of larval instars, prepupal, and pupal stages for each of the ten treatment combinations (aphid species + aphid density) for both the predators were noted. Larval survival and adult emergence were calculated using the following formulas:

$\text{Larval survival}(\%) = \frac{\text{Number of pupae formed}}{\text{Number of 1st instars used}}\times 100$

$\text{Adult emergence}(\%) = \frac{\text{Number of adults emerged}}{\text{Number of pupae used}}\times 100$

The surviving larvae and pupae were used for further investigation. The 4th instar larvae in the later part of their life became sluggish, suspended feeding, and stopped movements before ecdysis to pupae. The time period between inactivation and ecdysis to pupal stage was recorded as the prepupal period. The duration between ecdysis to pupa and emergence of adults was recorded as the pupal period. Additional cultures were used to harvest a sufficient number of adult females for each treatment combination. After eclosion, the adults were sexed and weighed. The longevity of both male and female adults was recorded as the time period between emergence of adults from the pupae and their death. Preoviposition period was worked out using mated females of both the predator species as the time period between adult emergence and initiation of egg-laying. Egg-laying by the mated females was observed daily, and the duration in days from initiation of egg-laying until its termination was recorded as the oviposition period. The time period from termination of egg-laying until the death of the mated females was recorded as the postoviposition period.

The cumulative number of eggs laid by a single female throughout its oviposition period was recorded as fecundity. During oviposition period, crumpled paper was added to the Petri dishes containing the mated females to provide oviposition sites. The paper was replaced daily, and the number of eggs laid was counted. The same eggs were used to work out the incubation period and hatchability percentage. The incubation period of the eggs, i.e., the time period elapsed between egg laying and hatching, was recorded for 100 eggs in each replication of all 10 treatment combinations for both the predator species. The number of eggs hatched was recorded daily until no more eggs hatched, and the weighted mean of eggs hatched per day and time period elapsed was calculated to work out the incubation period. The hatchability of eggs was determined as the percentage of eggs that hatched successfully out of the total number of eggs kept under observation (100 eggs per replication).

### Data analysis

The data on the duration of various growth stages, adult weight, larval survival, adult emergence, fecundity, and hatchability was subjected to analysis of variance (ANOVA) in factorial completely randomized design (CRD) for the significance of main effects (prey species and prey density) and interaction. Percentages of larval survival, adult emergence, and eggs hatched were transformed using arcsine transformation before analysis. Mean values were compared using least significant difference (LSD) test. The analysis was done using R-software (R Development Core Team 2008).

## Results

### Preadult development and survival

Both prey species (F = 101.20; d.f. = 1, 80; *P* < 0.0001) and prey abundance (F = 113.75; d.f. = 1, 80; *P* < 0.0001) were found to have a significant effect on the total larval period of *A. tetraspilota* as well as *H. variegata* (F = 209.3; d.f. = 1, 80; *P* < 0.0001 and F = 286.34; d.f. = 1, 80; *P* < 0.0001, respectively), as shown in Tables 1 and 2 . The total larval period was found to increase significantly when *A. pomi* was used as prey in substitution of *B. brassicae* for both the predator species. A greater effect of varying prey density was found on *H. variegata* , as the total larval period was noted as 13.27 days and 28.00 days at maximum and minimum prey densities tested, respectively. Variation in total larval period for *A. tetraspilota* was found to be significant over various prey abundance levels for a particular prey species as well as over the two prey species at each abundance level tested (F = 6.16; d.f. = 1, 80; *P* = 0.0002). However, the interaction effect was found to be insignificant for *H. variegata* (F = 2.13; d.f. = 1, 80; *P* = 0.0840).

**Table 1.**
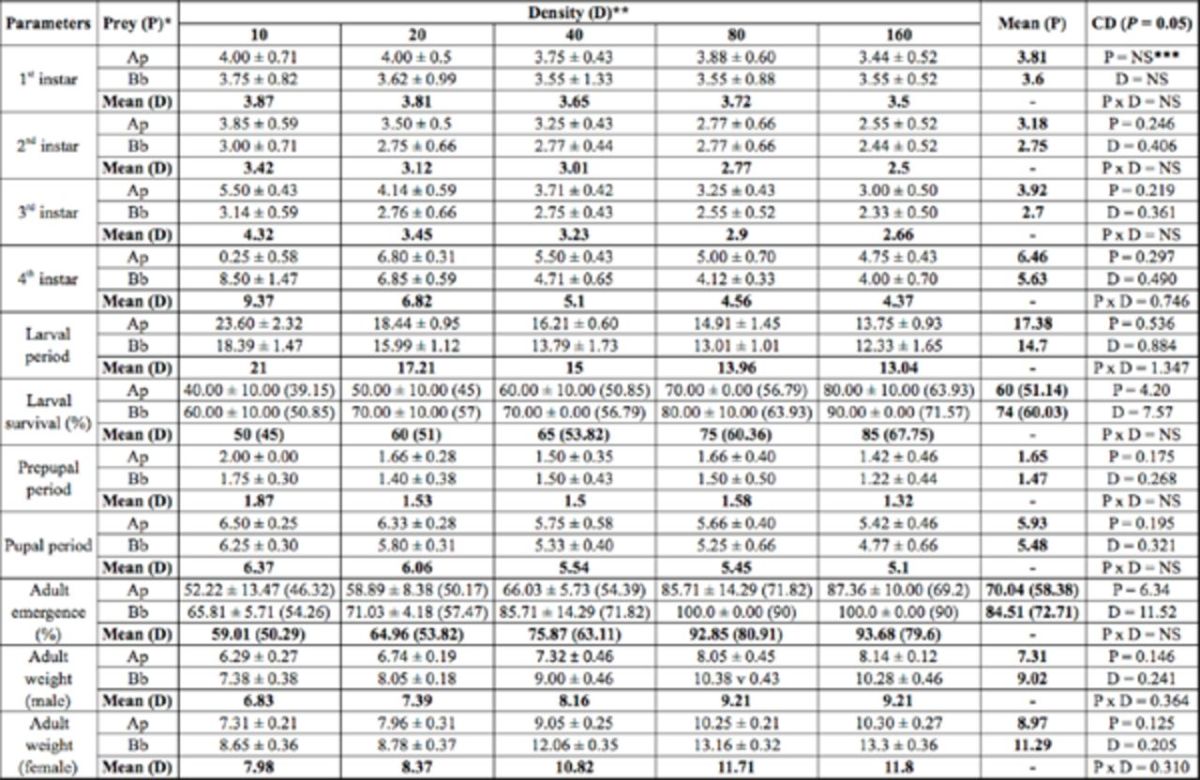
Developmental period (days), survival of immatures, and adult weight (mg) of *Adalia tetraspilota* that fed on two prey species and five prey densities.

Values are mean ± SE. Values in parenthesis are arcsine transformed values.

*Ap = *Aphis pomi* , Bb = *Brevicoryne brassicae*

** Number of aphids per day per predator

***NS = Non-significant

CD = Critical difference

For *A. tetraspilota* , the effect of prey species was found to be significant on 2nd (F = 12.81; d.f. = 1, 80; *P* = 0.0005), 3rd (F = 123.72; d.f. = 1, 80; *P* < 0.0001) and 4th instar larval duration (F = 30.93; d.f. = 1, 80; *P* < 0.0001). However, its effect was insignificant on 1 ^st^ instar duration (F = 1.607; d.f. = 1, 80; *P* = 0.2058). For *H. variegata* , prey species had a significant effect on all four larval instars. In all the cases, larval duration was longer when larvae fed on *A. pomi* as compared to *B. brassicae* . The effect of prey abundance followed the same trend for both the ladybird species, as the duration of each larval instar increased with decreases in prey density. The variation in duration was found to be insignificant at prey densities of 80 and 160 for all the larval instars of both the predators. For *A. tetraspilota* , the interaction effect of prey species and prey abundance was found to be significant for 4th instar larvae only (F = 3.77; d.f. = 4, 80; *P* = 0.0072). For *H. variegata* , the interaction effect was not found to be significant for any larval instar.

A significant variation in larval survival (%) when larvae fed on different prey species was noted for both *A. tetraspilota* (F = 20.60; d.f. = 1, 20; *P* = 0.0001) and *H. variegata* (F = 25.81; d.f. = 1, 20; *P* < 0.0001). Percent larval survival increased significantly when *B. brassicae* was used as prey as compared to when *A. pomi* was used for both the predators. The effect of prey abundance levels was also significant for both *A. tetraspilota* (F = 16.02; d.f. = 4, 20; *P* < 0.0001) and *H. variegata* (F = 15.16; d.f. = 4, 20; *P* < 0.0001). The interaction effect of prey species and prey abundance was insignificant for both *A. tetraspilota* (F = 0.404; d.f. = 4, 20; *P* = 0.8035) and *H. variegata* (F = 0.620; d.f. = 4, 20; *P* = 0.6530).

Prey species had a significant effect on the duration of prepupal (F = 4.87; d.f. = 1, 80; *P* = 0.0300) and pupal stages (F = 21.90; d.f. = 1, 80; *P* < 0.0001) for *A. tetraspilota* . The same trend was noted for *H. variegata* (F = 70.34; d.f. = 1, 80; *P* = 0.0096 for prepupal period, F = 63.78; d.f. = 1, 80; *P* < 0.0001 for pupal period). In both cases, the respective periods got extended appreciably when *A. pomi* was used as prey as compared to when *B. brassicae* was used. For *A. tetraspilota* , the effect of varying prey density was found to be significant on both the prepupal (F = 4.89; d.f. = 4, 80; *P* = 0.0013) and pupal period (F =21.89; d.f. = 4, 80; *P* < 0.0001). Similarly, for *H. variegata* , prey abundance was found to significantly affect the duration of the prepupal (F = 3.49; d.f. = 4, 80; *P* = 0.0100) and pupal (F = 118.69; d.f. = 4, 80; *P* < 0.0001) periods. For both predator species, the variation of pupal period over the prey densities of 160 and 80 was found to be insignificant. The interaction of prey species and prey density was insignificant for both prepupal and pupal periods for both predators.

The adult emergence percentage was found to be significantly affected by the prey species for both *A. tetraspilota* (F = 23.16; d.f. = 1, 20; *P* = 0.0001) and *H. variegata* (F = 7.75; d.f. = 1, 20; *P* = 0.0110). For *A. tetraspilota* , adult emergence percentage was significantly higher (84.51%) for individuals fed *B. brassicae* as compared to those fed *A. pomi* (70.04%). The same trend was noted for *H. variegata* , and the corresponding figures were registered as 74.48% and 65.37%, respectively. Variation in prey density also affected adult emergence percentage significantly (F = 18.27; d.f. = 4, 20; *P* < 0.0001 for *A. tetraspilota* , F = 9.85; d.f. = 4, 20; *P* = 0.0001 for *H. variegata* ). The interaction of prey species and prey density was insignificant for *A. tetraspilota* (F = 0.88; d.f. = 4, 20; *P* = 0.4920) but significant for *H. variegata* (F = 5.668; d.f. = 4, 20; *P* = 0.0032).

Analysis of variance showed a significant effect of prey species (F = 546.06; d.f. = 1, 90; *P* < 0.0001) and prey density (F = 170.16; d.f. = 4, 90; *P* < 0.0001) on body weight of adult males of *A. tetraspilota* . The weight was 19% higher for males fed *B. brassicae* compared to those fed *A. pomi* . Body weight of adult females also varied significantly with prey species (F = 1392; d.f. = 1, 90; *P* < 0.0001) and prey density (F = 694.72; d.f. = 4, 90; *P* < 0.0001). Adult females fed *B. brassicae* were 20.5% heavier than those fed *A. pomi* , which had a direct bearing on reproductive parameters. The interaction of prey species and prey density had a significant effect on body weight of both males (F = 10.42; d.f. = 4, 90; *P* < 0.0001) and females (F = 71.72; d.f. = 4, 90; *P* < 0.0001) of *A. tetraspilota* . For *H. var-iegata* , a significant effect of prey species (F = 120; d.f. = 4, 90; *P* < 0.0001 for males; F = 222.78; d.f. = 1, 90; *P* < 0.0001 for females) and prey density (F = 29.93; d.f. = 4, 90; *P* < 0.0001 for males; F = 156.54; d.f. = 4, 90; *P* < 0.0001 for females) on body weight of adults was found. The interaction of the two factors for adult weight was, again, significant for both males (F = 2.87; d.f. = 4, 90; *P* = 0.0272) and females (F = 10.14; d.f. = 4, 90; *P* < 0.0001). The variation of adult weight of males and females of both predator species was insignificant as prey density varied from 80 to 160 aphids per day.

### Reproduction and longevity

Data on the effect of prey species and prey density on various reproductive parameters and adult longevity of the two predator species are shown in Tables 3 and 4 .

**Table 2.**
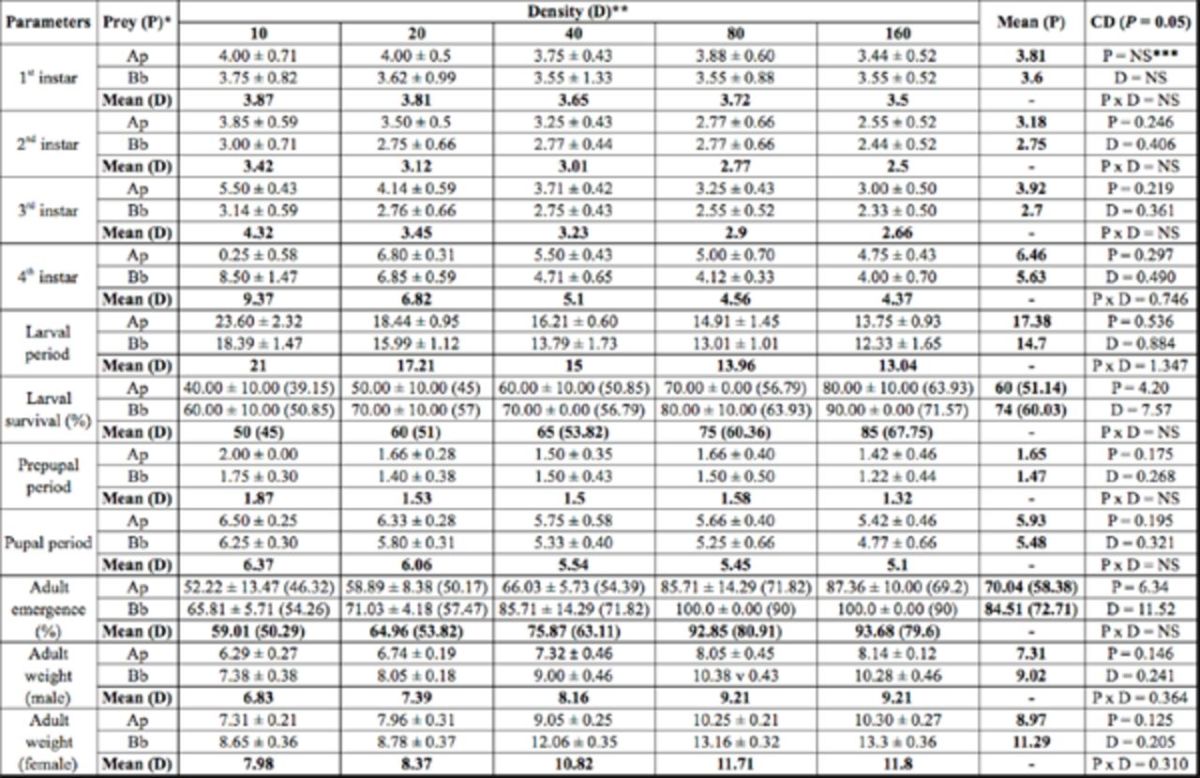
Developmental period (days), survival of immatures, and adult weight (mg) of *Hippodamia variegata* that fed on two prey species and five prey densities.

Values are mean ± SE. Values in parenthesis are arcsine transformed values.

*Ap = *Aphis pomi* , Bb = *Brevicoryne brassicae*

** Number of aphids per day per predator

***NS = Non-significant

CD = Critical difference

**Table 3.**
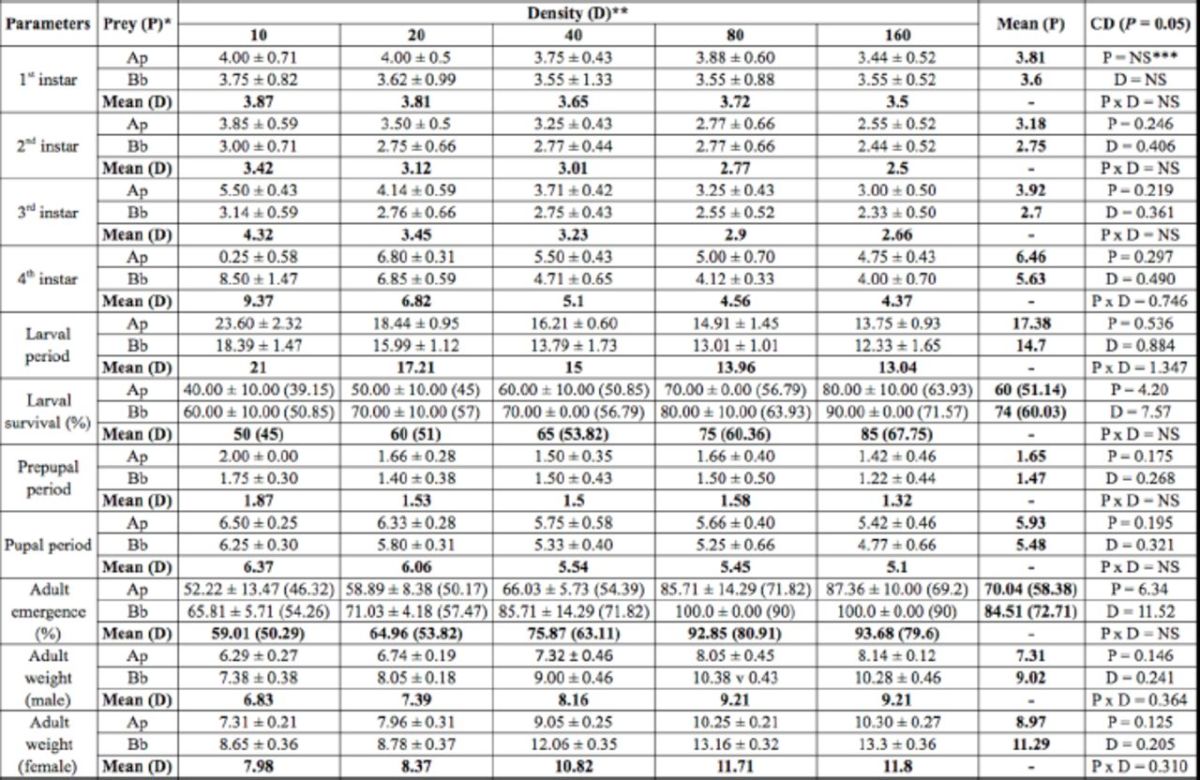
Reproductive parameters and adult longevity of *Adalia tetraspilota* on two prey species and five aphid densities.

Values are mean ± SE. Values in parenthesis are arcsine transformed values.

*Ap = *Aphis pomi* , Bb = *Brevicoryne brassicae*

** Number of aphids per day per predator

***NS = Non-significant

CD = Critical difference

**Table 4.**
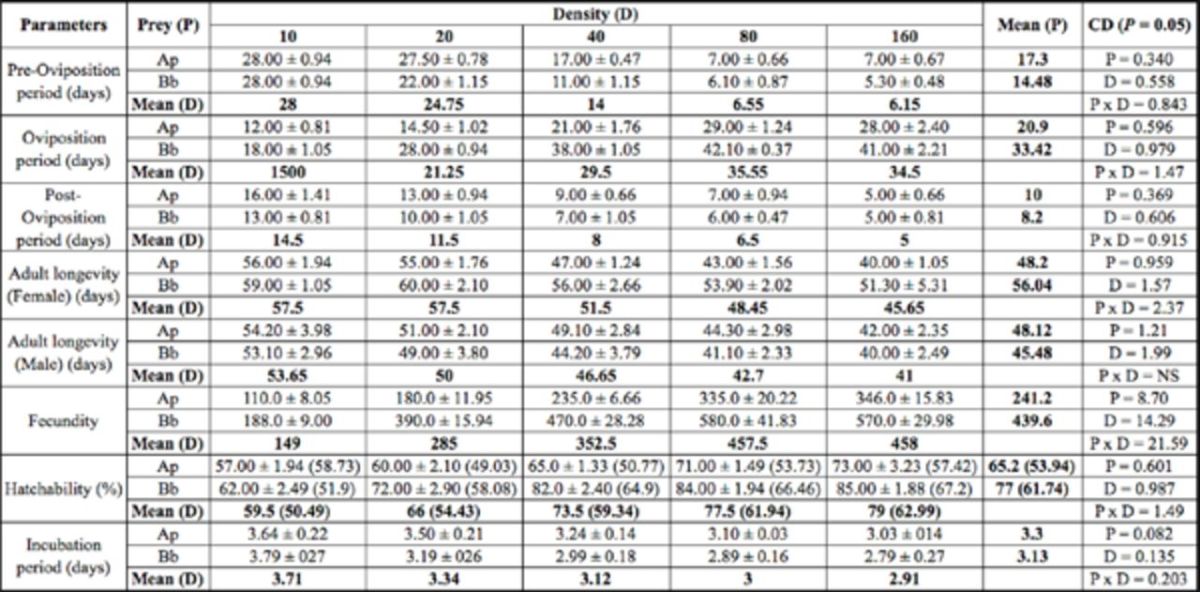
Reproductive parameters and adult longevity of *Hippodamia variegata* on two prey species and five aphid densities.

Values are mean ± SE. Values in parenthesis are arcsine transformed values.

*Ap = *Aphis pomi* , Bb = *Brevicoryne brassicae*

** Number of aphids per day per predator

***NS = Non-significant

CD = Critical difference

Both prey species and prey abundance influenced the preoviposition period of females for both predator species. Analysis of variance revealed a significant effect of prey species (F = 77.12; d.f. = 1, 90; *P* < 0.0001), prey abundance (F = 398.9; d.f. = 4, 90; *P* < 0.0001), and the interaction of prey species with prey abundance levels (F = 4.44; d.f. = 4, 90; *P* = 0.0025) on the preoviposition period for *A.tetraspilota* females. For *H. variegata* , both factors and their interaction were significant (F = 277.40; d.f. = 1, 90; *P* < 0.0001 for prey species, F = 2866.23; d.f. = 4, 90; *P* < 0.0001 for prey abundance, and F = 52.65; d.f. = 4, 90; *P* = 0.0047 for the interaction of the two). The variation in period length was found to be insignificant at the prey abundance levels of 80 and 160 for both predator species.

A significant variation was noted in the oviposition period on varying prey species as well as prey abundance. For *A. tetraspilota* , a significant effect of prey species (F = 1230.0; d.f. = 1, 90; *P* < 0.0001), prey abundance (F = 1075.65; d.f. = 4, 90; *P* < 0.0001), and the interaction of the two factors (F = 97.19; d.f.= 4, 90; *P* < 0.0001) on oviposition period was seen. The oviposition period was positively affected when *B. brassicae* was used as prey. For *H. variegata* , a significant effect of prey species (F = 1777.6; d.f. = 1, 90; *P* < 0.0001), prey abundance (F = 708.87; d.f. = 4, 90; *P* < 0.0001) and the interaction of the two factors (F = 36.35; d.f. = 4, 90; *P* < 0.0001) on the oviposition period of the females was found.

A significant variation in the postoviposition period due varying prey species and prey abundance was found for both predators. For *A. tetraspilota* , on varying the prey species (F = 74.70; d.f. = 1, 90; *P* < 0.0001), the postoviposition period decreased from 13.42 to 10.82 days when *A. pomi* was replaced by *B. brassicae* as prey. The postoviposition period was also found to be dependent on prey density (F = 352.48; d.f. = 4, 90; *P* < 0.0001). For *H. variegata* , a significant effect of prey species (F = 95.92; d.f. = 1, 90; *P* < 0.0001) and prey density (F = 353.48; d.f. = 4, 90; *P* < 0.0001) on postoviposition period was found. The interaction of prey species and prey abundance had a significant effect on postoviposition period for *H. variegata* (F = 10.06; d.f. = 4, 90; *P* < 0.0001) but an insignificant effect for *A. tetraspilota* (F = 0.59; d.f. = 4, 90; *P* = 0.6650). For both predators, the variation in the period was insignificant as the prey density decreased from 160 to 80 aphids per day per predator.

Adult longevity in both *A. tetraspilota* and *H. variegata* male and female individuals was found to be dependent on prey species and prey abundance. For *A. tetraspilota* , the analysis of variance revealed that prey species had a significant effect on longevity of adult males (F = 62.74; d.f. = 1, 90; *P* < 0.0001) and females (F = 88.06; d.f. = 4, 90; *P* < 0.0001). The adult females lived for 51.88 and 48.50 days when fed *B. brassicae* and *A. pomi,* respectively. In contrast, longevity of males was found to be lower for individuals fed *B. brassicae* (41.70 days) as compared to those that preyed upon *A. pomi* (46.42 days). Furthermore, prey abundance had a significant effect on the longevity of male (F = 42.46; d.f. = 4, 90; *P* < 0.0001) and female (F = 50.99; d.f. = 4, 90; *P* < 0.0001) individuals of *A. tetraspilota* . The longevity of both adult males and females increased with decreasing prey abundance. The interaction of prey species and prey abundance was insignificant for longevity of males (F = 0.578; d.f. = 4, 90; *P* = 0.6780) but significant for female individuals (F = 47.91; d.f. = 4, 90; *P* < 0.0001). For *H. variegata* , prey species had a significant effect on the longevity of both adult females (F = 269.58; d.f. = 1, 90; *P* < 0.0001) and males (F = 18.98; d.f. = 1, 90; *P* < 0.0001). Similarly, prey abundance also significantly affected the longevity of adult females (F = 99.65; d.f. = 4, 90; *P* < 0.0001) and males (F = 58.63; d.f. = 4, 90; *P* < 0.0001). Longevity of both adult females and males increased with decreases in prey density. The interaction of prey species and prey abundance had an insignificant effect on longevity of males (F = 1.17; d.f. = 4, 90; *P* = 0.3280) but a significant effect on longevity female individuals (F = 11.88; d.f. = 4, 90; *P* < 0.0001) of *H. variegata* . The longevity of adult females of both predator species was found to be statistically non-significant at the prey densities of 80 and 160 aphids per day. The same trend was noted for the longevity of adult males of both predator species.

For both predators, fecundity was found to be strongly influenced by quality and quantity of prey. For *A. tetraspilota* , analysis of variance revealed significant effects of prey species (F = 6647.8; d.f. = 1, 90; *P* < 0.0001) and prey abundance (F = 945.06; d.f. = 4, 90; *P* < 0.0001) on the fecundity of females. The females were found to lay more eggs when fed *B. brassicae* (435.8 eggs per female) as compared to when they were fed *A. pomi* (217.4 eggs per female). The fecundity decreased from 422 to 205.9 eggs as the prey abundance decreased from 160 to 10 aphids per day, however the interaction effect was insignificant (F = 1.99; d.f. = 4, 90; *P* = 0.1026). For *H. variegata* , a significant effect of prey species (F = 2095.34; d.f. = 1, 90; *P* < 0.0001) and prey abundance (F = 717.48; d.f. = 4, 90; *P* < 0.0001), as well as a significant interaction effect (F = 50.03; d.f. = 4, 90; *P* < 0.0001) on the fecundity of females was found. Variation in fecundity was found to be statistically insignificant between the prey densities of 80 and 160 aphids per day.

Just like most other characters, hatchability of eggs for both the predators was found to be dependent on prey species and prey abundance. For *A. tetraspilota* , hatchability (%) varied from 43.00% to 82.00%. Maximum hatchability was noted for eggs laid by females fed *B. brassicae* at the abundance level of 160 aphids per day, and the lowest hatchability was found for eggs laid by females fed *A. pomi* at the lowest prey abundance level, i.e.,10 aphids per day. For *H. variegata* , hatchability (%) was found to vary from 57.00 to 85.00. Lowest hatchability was noted for the eggs laid by females fed *A. pomi* at an abundance level of 10 aphids per day (lowest prey density), and highest hatchability was found on the eggs laid by the females fed *B. brassicae* at the highest abundance (160 aphids per day). Hatchability was found to be better for eggs of *H. variegata* as compared to those of *A. tetraspilota* under the same prey species and prey abundance conditions.

The incubation period of eggs of *A. tetraspilota* and *H. variegata* varied significantly with different prey species (F = 237.25; d.f.= 1, 90; *P* < 0.0001 and F = 18.06; d.f.= 1, 90; *P* < 0.0001, respectively). For *A. tetraspilota* , the incubation period was noted as 3.67 days and 4.02 days when *B. brassicae* and *A. pomi* were used as prey, respectively. The corresponding figures for *H. variegata* were noted as 3.13 and 3.30 days, respectively. The effect of varying prey abundance was also significant on incubation period for both *A. tetraspilota* (F = 153.02; d.f. = 4, 90; *P* < 0.0001) and *H. variegata* (F = 49.53; d.f. = 4, 90; *P* < 0.0001). The incubation period of *A. tetraspilota* eggs increased by 21% and those of *H. variegata* by 27% as the prey abundance decreased from 160 to 10 aphids per day. The interaction effects were noted to be significant for both predators (F = 24.77; d.f. = 4, 90; *P* < 0.0001 for *A. tetraspilota* , and F=4.02; d.f. = 4, 90; *P* = 0.0047 for *H. variegata* ).

## Discussion

The original assumption that all aphids are suitable for all species of aphidophagous coccinellids was rejected by Hodek (1960) and Blackman (1967) . Since then, it has generally been accepted that not all prey eaten are suitable food for coccinellids. The food enabling development and oviposition (essential prey) should be discriminated from the food that is good only for survival (alternative). Also, there is a category of rejected prey that may be toxic. However, there are different levels of suitability of individual essential preys ( Hodek and Honek 1996 ). The current investigation indicated that both *Aphis pomi* and *Brevicoryne brassicae* are essential prey for both *A. tetraspilota* and *H. variegata* , *B. brassicae* being more suitable in comparison to *A. pomi* .

The study also revealed that the coccinellid predators studied could complete their life cycle at the least prey density of 10 aphids per day per individual. This finding may be ascribed to the fact that predaceous coccinellids show a pronounced ability to adjust to food scarcity as an adaptation to intermittent absences of prey. Most of the biological parameters showed no significant variation as the prey abundance was increased from 80 to 160, indicating that the prey sufficiency lies below 80 aphids per predatory stage per day. Studies on functional response of the two predators on the same prey species ( Shah and Khan 2013 ) indicated that the satiation plateau is reached as the prey density increases from 40 to 80. Thus, the zone of prey sufficiency must lie between 40 to 80 aphids per predator per day. According to Hodek and Honek (1996) , the larvae of *Coccinella septumpunc- tata* (L.) could complete development when the food supply was artificially reduced to 55 or 40%, although the immature survival is considerably reduced and reproductive output is affected. Similar results were obtained from the current study. The effect of prey species and prey abundance on the various biological parameters of *A. tetraspilota* and *H. variegata* is discussed below.

### Preadult development and survival

The durations of all larval instars of *H. variegata* and all but the first instar of *A. tetraspilota* were found to vary significantly with prey species and prey abundance, thus affecting the total larval period of both the predators. The larval duration was consistently longer in individuals that fed on *A. pomi* as compared to those that fed on *B. brassicae* . According to Shafiei et al. (2001) , lengthening of the developmental period is a mechanism that allows insects to survive inadequate nutrition during the larval stage, as it allows insects to extend their feeding activity so as to acquire enough food resources to complete growth. Varied palatability of the two aphid prey species for the coccinellid predators may be attributed to the species specific alkanes present on the surface of the aphids ( Liepert and Dettnere 1996 ) and differences in the wax patterns of the aphids that could be used in the recognition and determination of palatability ( Kosaki and Yamaoka 1996 ). Higher palatability of *B. brassicae* for *A. tetraspilota* and *H. variegata* larvae may be attributed to their nutrient contents, which probably ease digestion (Pervez and Omkar 2004 ). Reduced consumption of some aphids has been ascribed to certain alkaloids and other allelochemicals not suitable for the constitution and metabolism of the ladybeetles ( Okamoto 1966 ). However, this aspect needs to be confirmed by chemical analysis of body contents. The reduced consumption in response to chemical constituents might maintain the unwanted chemicals below harmful levels but still ensure survival, thus it became clear that *B. brassicae* is a more suitable prey for both *A. tetraspilota* and *H. variegata* as compared to *A. pomi* .

The larval period lasted for 20.91 and 16.07 days on *A. pomi* and *B. brassicae* , respectively, for *H. variegata* , and 17.38 and 14.7 days, respectively for *A. tetraspilota* . No reports were found regarding the biology of *A. tetraspilota* ; however sufficient literature exists regarding the various biological aspects of *H. variegata* . The larval duration has been reported as 16.5 days for *H. variegata* reared on *Aphis fabae* ( Jafari 2011 ), 8.83 days for *H. variegata* larvae reared on *Aphis gossypii* ( Wu et al. 2010 ), 11.8 days for *H. variegata* reared on *B. brassicae* and *Rhopalosiphum padi* ( ElHag and Zaitoon 1996 ), and 9.4 days for *H. variegata* reared on *Myzus persicae* ( Lanzoni et al. 2004 ). Variation in the presented findings may be ascribed to factors such as prey species, host plant of prey, rearing conditions, etc., that are reported to affect various biological parameters ( Lanzoni et al. 2004 ). Besides, geographical variations in the predator-prey system may also be important ( Dobzhansky 1933 ).

The prey abundance levels significantly affected the larval period of both predators. For, *A. tetraspilota* , the larval period was limited to 13.04 days at the highest prey density (160) and extended to a period of 21.0 days at the lowest prey density (10). For *H. variegata* , the larval period increased from 13.27 days to 28 days as the prey density decreased from 160 to 10. The elongation of developmental periods with shortage of food has been reported for many ladybeetle species, such as *Coloe- omegilla maculata* ( Cividanes et al. 2010 ), *Harmonia axyridis* ( Agarwala et al. 2008 ), *Harmonia dimidiata* ( Sharmila et al. 2010 ), *Adalia bipunctata* ( Wratten 1973 ), *Propylea japonica* (Kauwachi 1979), *Coleomegilla maculata* , *Hippodamia convergens* and *Harmonia axyridis* ( Phoofolo et al. 2008 ). According to Schuder et al. (2004), ladybeetles react to a shortage of food by developing more slowly.

Insignificant variation in the larval duration of *H. variegata* when the prey abundance decreased from 160 to 80 indicated that the zone of food sufficiency for *H. variegata* might be close to 80 aphids per day per predator. According to Dixon (2000) , there is definite quantity of food that acts as threshold above which the developmental rate is optimal.

Appreciable variation in survivorship of larvae was noted by varying the prey species and prey abundance. For both the predators, larval survival was higher for individuals reared on *B. brassicae* as compared to those reared on *A. pomi* . Mortality was noted to increase as the prey abundance decreased. Larval survival decreases as less of the prey is consumed, either due to its less suitability or shortage in supply (Kaluskov and Hodek 2004). A similar effect of varying prey species and prey abundance was noted on the adult emergence percentage of both the predator species. The pupae suffered more mortality on *A. pomi* as compared to *B. brassicae* . The emergence percentage consistently increased with increasing prey abundance levels. According to Parvez and Omkar (2004) , possible reasons for increased mortality of immatures on the less consumed prey include slow starvation resulting from lower consumption and/or inability of the ladybird metabolism to detoxify or sequester the unsuitable chemicals. In general, neonate larvae and 4th instars suffered the maximal mortality. First instars suffered higher mortality because of their thin cuticle making them more vulnerable to physical stresses ( Ponsonby and Copland 1996 ). The 4th instar larvae suffered more as the prey abundance decreased, as the higher metabolic needs could not be catered to ( Cividanes et al. 2010 ). Larval survival in the range of 50–75% has been reported for *H. variegata* by different studies ( ElHag and Zaitoon 1996 ; Lanzoni et al. 2004 ; Wu et al. 2010 ). Wu et al. (2010) reported the adult emergence of *H. variegata* fed *A. gossypii* , reared on five host plants, varied from 87.23 to 100%. Overall, preadult survival was found to vary from 44.06 to 58.97% depending on the host plant of the prey. Lanzoni et al. (2004) reported the immature survival of *H. variegata* reared on *M. persicae* as 49.1%, while ElHag and Zaitoon (1996) reported 61.8% immature survival of *H. variegata* reared on *B. brassicae* .

The prepupal and pupal periods of both the predators increased significantly as *A. pomi* was used as prey as compared to *B. brassicae* . The effect of varying prey species on prepupal and pupal periods is known for many other coccinellid predators, such as *C. septumpunc- tata* ( Omkar and Srivastava 2003 ; Kalushkov and Hodek 2004 ), *A. bipunctata* ( Jalali et al. 2009 ), *C. sexmaculata* ( Omkar and Bind 2004 ), and *P. dissecta* (Pervez and Omkar 2004 ), to mention a few. The effect of varying prey abundance levels was also found to be significant on the prepupal and pupal periods of both *A. tetraspilota* and *H. variegata* ; increasing prey density decreased the respective developmental periods. Sharmila et al. (2010) reported a significant effect of prey density on prepupal and pupal period of *H. dimidiata* . Similar effects have earlier been reported by Kawauchi (1979) on *P. japonica* and Hukusima and Ohwaki (1972) on *H. axyridis* . A significant effect of prey density on pupal period of *H. axyridis* has also been reported by Agarwala et al. (2008) . The pupal period of *H. variegata* varied from 5.11 to 7.75 days on feeding upon different prey species and under different prey abundance conditions. The values are in close proximity of earlier reported values from ElHag and Zaitoon (1996) , Lanzoni et al. (2004), Wang et al. (2004) , Rebolledo et al. (2007), and Jafari (2011) .

The highest fresh weight of adult ladybird beetles was attained after feeding on *B. brassicae* , and this confirms its suitable nutritive content, as indicated by the variation in the duration of various developmental stages with prey species. The weight of adults was appreciably lower when the prey density decreased from 160 to 10 aphids per day. The variation in body weight of adults had direct bearing on various reproductive parameters, most notably on fecundity and oviposition period of females, as discussed below.

### Reproductive parameters

Both the reproductive (oviposition) and non- reproductive (pre- and postoviposition) periods of adult females were found to be dependent on prey species and prey abundance. The longest reproductive periods with shortest non-reproductive periods were noted for females fed *B. brassicae* as compared to those fed *A. pomi* . This finding confirms the relatively higher suitability of *B. brassicae* as prey for both *A. tetraspilota* and *H. variegata* . The non-reproductive periods increased significantly while the reproductive periods decreased with decreasing prey abundance.

Omkar and Srivastava (2003) reported that increased quantity of high quality food decreased the length of preoviposition period. The decreased consumption of less suitable foods, by affecting the preadult development, probably results in slower sexual maturation and longer preoviposition periods (Kauwachi 1981). High consumption of suitable prey supports early ovariole maturation and provides energy and nutriment to sustain a longer oviposition period, and vice versa for less suitable or unsuitable food ( Honek 1980 ). The females with long reproductive periods result in higher reproductive output (fecundity). Several reports suggest a tradeoff between adult longevity and fecundity ( Dixon 2000 ). Hence, the females with long oviposition periods have relatively shorter postoviposition periods.

The preoviposition, oviposition, and postoviposition periods were noted as 12.6, 22.92, and 10.82 days on *A. pomi* , and 9.96, 31.10, and 13.42 days on *B. brassicae* , respectively for *A. tetraspilota* females. For *H. variegata* females, preoviposition period was noted as 17.3 and 14.48 days, oviposition period as 20.9 and 33.42 days, and postoviposition period as 10.0 and 8.2 days, respectively on *A. pomi* and *B. brassicae* . For *H. variegata* , the preoviposition period has been reported as 6.2 days ( Jafari 2011 ), 6.5 days ( ElHag and Zaitoon, 1996 ), 7 days ( Lanzoni et al. 2004 ), and 3.82 days ( Wu *et* al., 2010 ) for adult females reared on different prey species. Wu et al. (2010) reported the oviposition and postoviposition period of 30.53 and 6.18 days, respectively, for *H. variegata* females reared on *A. gossypii* . Lanzoni et al. (2004) reported a mean oviposition period of 32.2 days for *H. variegata* feeding on *M. persicae* , while Jafari (2011) reported the period to vary from 37 to 48 days for *H. variegata* females reared on *A. fabae* . Discrepancies in experimental methods, including different rearing conditions, may help to explain these variations. Furthermore, geographical variability produces differences in various biological attributes of the coccinellid predators (Bobzhansky 1933).

Reproductive output was appreciably higher for females fed *B. brassicae* (more suitable prey), and the fecundity drastically decreased under food shortage conditions. Higher fecundity for females reared on more suitable and abundant aphid species has been reported to be due to increased prey consumption leading to higher conversion of food to eggs (Baumgartner et al. 1987; Pervez and Omkar 2004 ). Kalushkov and Hodek (2004 ) reported that essential aphid foods affected adult weight at eclosion and hence fecundity of ladybeetles.

*A. tetraspilota* females laid 217 and 435.8 eggs on average when reared on *A. pomi* and *B. brassicae* , respectively. The females of *H. variegata* were found to lay an average of 241.2 and 439.6 eggs, respectively on *A. pomi* and *B. brassicae* . Lanzoni et al. (2004) reported a mean fecundity of 841.7 eggs for *H. variegata* females reared on *M. persicae* . Kontodimas and Stathas (2005) reported the fecundity as 276.3 eggs; Wu et al. (2010) reported the fecundity as 647.58 eggs, while Jafari (2011) reported a mean fecundity of 943.9 eggs for *H. variegata* females. The variation in reported figures of fecundity may be due to variation in prey species used, variation in nutritional quality of prey, or the rearing conditions (Kaluskov and Hodek 2004).

Hatchability of eggs was found to be strongly dependent on feeding history of the parents, as hatchability percentage varied significantly with prey species and prey abundance. Again, egg hatchability was significantly higher on *B. brassicae* as compared to *A. pomi* . The hatchability declined as the prey abundance was reduced. Simmons (1988) reported high consumption of suitable prey to increase the weight of eggs, which contained a large quantity of yolk and consequently increased egg viability.

The hatching percentage noted for *A. tetraspilota* was 59.8 and 69.4% on *A. pomi* and *B. brassicae* , respectively. For *H. variegata* eggs, the corresponding figures were noted as 65.2 and 77.0%, respectively. Wu et al. (2010) reported the hatchability as 85.9% for the eggs of *H. variegata* reared on *A. gossypii* . Jafari (2010) reported that 82.86% of *H. variegata* eggs hatched, while Elhabi et al. (2000) reported a mean hatchability of 79% for the same species. Lanzoni et al. (2004) reported that 70% of the *H. variegata* eggs hatched successfully. The reports still confirm that egg hatchability is dependent on the prey used and rearing methodology adopted.

Incubation period of the eggs laid by females that were fed different prey species in definite quantities showed significant variation. The eggs of the parents that were fed *B. brassicae* hatched earlier compared to those fed *A. pomi* . Such an effect of parental diet on the embryo- genesis has been reported by many workers, like Pervez and Omkar (2004) for *P. dissecta* , Kaluskov and Hodek (2004) for *C. sep- tumpunctata* , and Jalali et al. (2009) for A *. bipunctata* . The incubation period of *H. variegata* eggs has been reported to last for 3.35 days ( Jafari 2011 ), 2.6 days ( Lanzoni et al. 2004 ), 2.42 days ( Wu et al. 2010 ), and 2.8 days ( ElHag and Zaitoon 1996 ) under different rearing conditions on different preys.

### Adult longevity

The variation of prey species and prey abundance significantly affected the longevity of both male and female adults. In both cases, the females lived longer than the males. The *A. tetraspilota* females lived for 48.5 and 51.88 days when reared on *A. pomi* and *B. brassicae* , respectively. The *H. variegata* females lived for 48.2 and 56.04 days when fed upon *A. pomi* and *B. brassicae* , respectively. Wu et al. (2010) reported a longevity of 38.95 days for adult females reared on *A. gossypii* . Jafari (2011) reported an average longevity of 55.5 days for adults of *H. variegata* reared on *A. fabae* , while Rebolledo et al. (2007) reported a mean longevity of 55.09 days for adult females of *H. variegata* . The adult males of *A. tetraspilota* were found to live for 46.42 and 41.7 days when reared on *A. pomi* and *B.brassicae* , respectively, while the adult males of *H. variegata* lived for 48.12 and 45.48 days on the two prey species, respectively. Rebolledo et al. (2007) reported a mean longevity of 51.45 days for adult males of *H. variergata* , while Wu et al. (2010) reported the male longevity as 36.21 days. ElHag and Zatoon (1996) reported a mean adult longevity of 71.8 days for *H. variegata* . Elhabi et al. (2000) found that the longevity of male and female adults was 44.0 and 61.0 days respectively for *H. variegata* .

The adult females of both the predators lived longer when they fed on *B. brassicae* . This result is because of the elongated oviposition period, as reported for many other ladybeetles such as *C. septempuncata* ( Kalushkov and Hodek 2004 ), *A. bipunctata* ( Jalali et al. 2009 ), *P. dissecta* ( Pervez and Omakar 2004 ), and others. The longevity of both male and female adults increased with decreasing prey availability. This result can be explained as an adaptation to food shortage and reproduction- longevity tradeoff for females. Ohgushi (1996 ) reported that the tradeoff becomes more apparent when the organisms are food limited. Decreasing the prey abundance reduced the fecundity, hence the longevity increased.

The study confirms that both *A. pomi* and *B. brassicae* are suitable prey for both *A. tetraspilota* and *H. variegata* , the latter prey species being comparatively more suitable. The prey density for optimum growth and development lies in the range of 40 to 80 per predator per day for 3rd and 4th instar nymphs.
